# Global molecular diversity of RSV – the “INFORM RSV” study

**DOI:** 10.1186/s12879-020-05175-4

**Published:** 2020-06-26

**Authors:** Annefleur C. Langedijk, Robert Jan Lebbink, Christiana Naaktgeboren, Anouk Evers, Marco C. Viveen, Anne Greenough, Terho Heikkinen, Renato T. Stein, Peter Richmond, Federico Martinón-Torres, Marta Nunes, Mitsuaki Hosoya, Christian Keller, Monika Bauck, Robert Cohen, Jesse Papenburg, Jeffrey Pernica, Marije P. Hennus, Hong Jin, David E. Tabor, Andrev Tovchigrechko, Alexey Ruzin, Michael E. Abram, Deidre Wilkins, Joanne G. Wildenbeest, Leyla Kragten-Tabatabaie, Frank E. J. Coenjaerts, Mark T. Esser, Louis J. Bont

**Affiliations:** 1 Department of Paediatric Immunology and Infectious Diseases, Wilhelmina Children’s Hospital, University Medical Centre Utrecht, Utrecht University, Utrecht, the Netherlands; 2 Department of Medical Microbiology, University Medical Center Utrecht, Utrecht, the Netherlands; 3 Julius Center for Health Sciences and Primary Care, University Medical Center Utrecht, Utrecht, the Netherlands; 4 Department of Women and Children’s Health, School of Life Course Sciences, Faculty of Life Sciences and Medicine, King’s College London, London, UK; 5 ReSViNET foundation, Zeist, the Netherlands; 6 Department of Paediatrics, University of Turku and Turku University Hospital, Turku, Finland; 7 Centro INFANT at Pontificia Universidade Catolica de Rio Grande do Sul, Porto Alegre, Brazil; 8 Department of Paediatrics, The University of Queensland, Brisbane, Australia; 9 Department of Paediatrics, Hospital Clínico Universitario de Santiago, Santiago, Galicia Spain; 10 Medical Research Council: Respiratory and Meningeal Pathogens Research Unit, School of Pathology, Faculty of Health Sciences, University of the Witwatersrand, Johannesburg, South Africa; 11 Department of Science and Technology/National Research Foundation: Vaccine Preventable Diseases Unit, Faculty of health Sciences, University of the Witwatersrand, Johannesburg, South Africa; 12 Department of Paediatrics, Fukushima Medical University School of Medicine, Fukushima, Japan; 13 Department of Virology, University Hospital Giessen and Marburg, Marburg, Germany; 14 Department of Paediatrics, University Hospital Giessen and Marburg, Marburg, Germany; 15 Association Clinique et Thérapeutique Infantile du Val-de-Marne, CHI Créteil, GRC Gemini, Université Paris XII, Créteil, France; 16 Department of Paediatrics, Division of Pediatric Infectious Diseases, Montreal Children’s Hospital, McGill University Health Centre, Montreal, Canada; 17 Department of Paediatrics, McMaster University, Hamilton, Canada; 18 Paediatric Intensive Care Unit, Wilhelmina Children’s Hospital, University Medical Centre Utrecht, Utrecht University, Utrecht, the Netherlands; 19 AstraZeneca, Gaithersburg/South San Francisco, USA; 20 Julius Clinical, Zeist, the Netherlands

**Keywords:** Respiratory syncytial virus, Next generation sequencing, Temporal and geographical diversity, Molecular epidemiology, Monoclonal antibodies, Vaccines

## Abstract

**Background:**

Respiratory syncytial virus (RSV) is a global cause of severe respiratory morbidity and mortality in infants. While preventive and therapeutic interventions are being developed, including antivirals, vaccines and monoclonal antibodies, little is known about the global molecular epidemiology of RSV. INFORM is a prospective, multicenter, global clinical study performed by ReSViNET to investigate the worldwide molecular diversity of RSV isolates collected from children less than 5 years of age.

**Methods:**

The INFORM study is performed in 17 countries spanning all inhabited continents and will provide insight into the molecular epidemiology of circulating RSV strains worldwide. Sequencing of > 4000 RSV-positive respiratory samples is planned to detect temporal and geographical molecular patterns on a molecular level over five consecutive years. Additionally, RSV will be cultured from a subset of samples to study the functional implications of specific mutations in the viral genome including viral fitness and susceptibility to different monoclonal antibodies.

**Discussion:**

The sequencing and functional results will be used to investigate susceptibility and resistance to novel RSV preventive or therapeutic interventions. Finally, a repository of globally collected RSV strains and a database of RSV sequences will be created.

## Article summary

### Strengths

- INFORM RSV is large enough to identify drivers of spatial and temporal distribution.
- Sequencing platform was selected based on a comparative pilot study.
- RSV is cultured to translate genotype to function.
- INFORM RSV is collaborating with others including researchers from the UEDIN, WHO and NIH.

### Limitations

- Clinical information is limited, no follow-up data available.

## Background

Respiratory syncytial virus (RSV) is the leading cause of lower respiratory tract infections in children worldwide [1]. While most children infected with RSV suffer from runny noses, coughing and wheezing, RSV infection can escalate to bronchiolitis, pneumonia and even death [2]. Globally in 2015, 48,000–74,500 children under the age of 5 years died with RSV in-hospital, predominantly in low- and middle-income countries [2].

Although RSV is recognized as a global health problem, there is no licensed vaccine currently available anywhere in the world. Efforts to develop a vaccine initially failed in the 1960s when the first vaccine candidate, a formalin-inactivated vaccine, did not protect against RSV in children but instead induced exacerbated lung disease after subsequent RSV exposure requiring hospitalization and causing death [3, 4]. The potential risk of enhanced disease has hampered vaccine development such that, even after more than 50 years of effort, no vaccine is available yet. An alternative approach for prevention of RSV disease is passive immunization with monoclonal antibodies (mAbs). RSV-IGIV (RespiGam), an intravenous immunoglobulin containing high titers of RSV neutralizing antibodies, was initially approved in 1995 as a passive immunization strategy but was discontinued in 2003 after its replacement by the more potent mAb palivizumab (humanised mAb that targets the RSV fusion (F) protein) [5]. Palivizumab is the only currently approved prophylaxis and its use is limited to high-risk infants (premature, heart and lung disease, Down syndrome) in high-income countries [3]. These data demonstrate that neutralizing Abs are efficient in preventing RSV disease and that antibody levels correlate with RSV disease prevention. The development of suptavumab (REGN2222), another mAb targeting the RSV F protein as a preventive strategy for use in preterm infants was discontinued in 2017 as it failed to meet the primary endpoint of preventing medically-attended RSV infections [6, 7]. A promising mAb candidate currently in clinical development is nirsevimab (MEDI8897), which targets the prefusion form of RSV F protein [8]. With a higher potency and extended half-life as compared to palivizumab, nirsevimab holds promise for protecting from RSV-associated lower respiratory disease for all infants entering their first RSV season and high-risk infants entering their first and second RSV seasons [7, 8].

Future clinical use of therapeutics, vaccines and mAbs to prevent RSV raises concerns about the emergence of local resistant strains [9, 10]. Therefore, RSV global surveillance is required. The Observational US Targeted Surveillance of Monoclonal Antibody Resistance and Testing of RSV (OUTSMART-RSV) surveillance program characterized circulating RSV strains in the U.S. during the 2017–18 season [11]. RSV strains that are resistant to palivizumab were found to be rare [10]. The frequency of natural resistance-associated polymorphisms for nirsevimab was also low (in vitro < 1%). However, the degree to which the acquisition of resistance will impact the effectiveness of current and future RSV therapeutics on a global scale remains unclear. To date, mAb-resistant mutants (MARMs) have not been thoroughly studied worldwide and little is known about the prevalence of naturally occurring resistant RSV strains either. The International Network For Optimal Resistance Monitoring of RSV (INFORM RSV) study will therefore prospectively describe the molecular epidemiology of RSV by monitoring temporal and geographic distribution of whole viral genome sequences. In addition to monitoring, we will construct a large repository of RSV sequence derived from a diverse geographic location. In the present article, we describe the methodology of the INFORM RSV study.

### Study objectives

#### Primary objective

To investigate the molecular diversity of RSV isolates recovered from a global population of children less than 5 years of age over a five-year period.

#### Secondary objectives

1. To evaluate the prevalence of strains with polymorphisms in the binding regions for RSV mAbs
2. To compile a repository for RSV sequences
3. To perform functional virology studies
4. To test for susceptibility of newly identified RSV strains to RSV mAbs
5. To establish natural molecular evolution of RSV genomes before the widespread use of RSV mAbs or vaccines

## Methods

### Study design

INFORM RSV is a global clinical study initiated in 2017 by AstraZeneca to prospectively analyze RSV strains collected from children < 5 years of age. Collaborators were identified via the Respiratory Syncytial Virus Network (ReSViNET; www.resvinet.org). The ReSViNET Foundation is the international leading non-profit organization committed to reducing global burden of RSV infection. In the INFORM RSV study, RSV positive nasal samples will be collected from subjects as part of routine clinical care at local clinical sites and shipped to the laboratory of the University Medical Centre Utrecht (UMCU), the Netherlands, for sequencing and culturing.

**Fig. 1:**
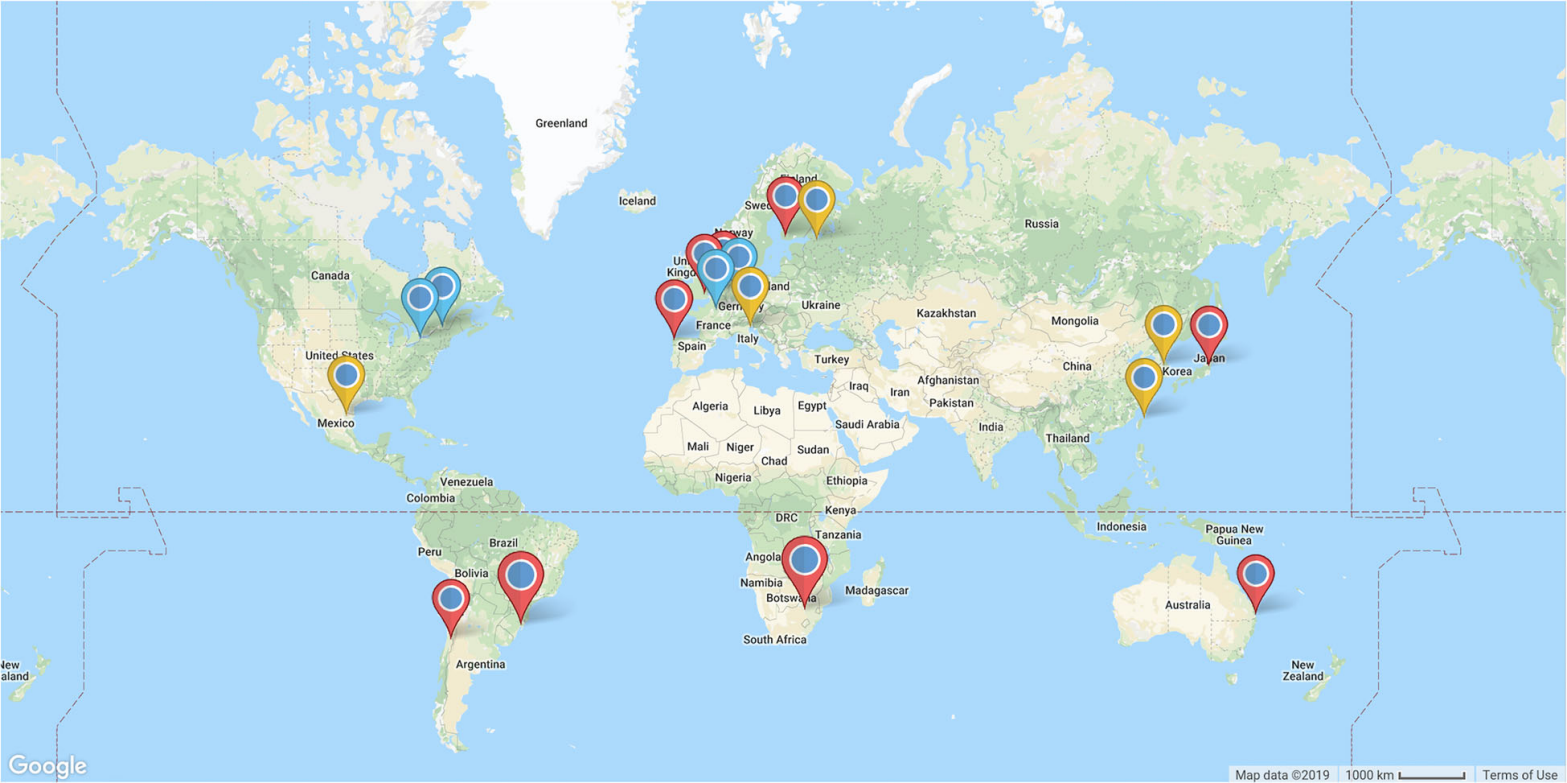
Countries participating in the INFORM RSV study. Red – Start in 2017; Blue – Start in 2018; Yellow – Start in 2019. The figure was created by ACL using Maptive (https://www.maptive.com)

In the INFORM RSV study, the goal is to collect and sequence approximately 4000 RSV positive respiratory samples during a 5-year period (2017–2022), which correlates to 50 or 100 samples per site per year (Additional files, Table 1). At the time of writing, the INFORM RSV study has been ongoing for 2 years and is currently conducted in 17 countries at 18 sites (Fig. 1). We aim to expand to other countries where disease burden studies are ongoing. To ensure both seasonal and geographical diversity, we endeavor to collect 10–20 samples per site per month, over the ~ five-month RSV season, which is on average 5 months long. If the site is able to collect more than the required number of samples, a subset will be randomly selected. Viral genomic sequencing will be performed on all samples by NGS using RT-PCR amplified cDNAs at the UMCU laboratory. To study molecular resistance, a subset of strains (~ 10%) will be randomly selected and cultured to evaluate functional susceptibility to anti-viral drugs being developed, and viral fitness of RSV variant with drug binding site changes or dominant changes in non-drug binding site.

### Study participants

Children are eligible to participate in the study if they meet all the inclusion criteria (Table 1). Children can participate in the study when they fulfill all the following criteria: (1) under the age of 5 years at time of sampling, (2) admitted to the hospital or visiting the outpatient clinic, (3) tested positive for RSV or suspected to have RSV infection when RSV testing is not standard practice. Suspicion of RSV is defined by respiratory tract infection (RTI) symptoms. In instances where testing for RSV is not a standard of care, the informed consent procedure is performed before sample collection for study purposes. Lower and upper RTIs are not differentiated. Signed and dated written informed consent is obtained from parent(s)/legal representative(s) in accordance with the INFORM RSV study protocol, the International Conference on Harmonization Guideline on Good Clinical Practice E6 (ICH-GCP) and applicable national and international regulatory requirements including the Declaration of Helsinki. Children who meet the exclusion criteria of using preventive or treatment medication for RSV e.g. palivizumab, ribavirin or an experimental RSV mAb or vaccine will be excluded from participation.

**Table 1:** Eligibility criteria for the INFORM RSV study

Inclusion Criteria	Exclusion Criteria
Age < 5 years	Use of palivizumab or experimental medication for RSV
Confirmed RSV positive diagnosis	
Written informed parental consent	

### Sample collection, storage and shipment

After informed consent is obtained, nasopharyngeal samples are collected using flocked swabs and placed in Copan Universal Transport Medium (UTM). When patients are ventilated, bronchial aspirates are collected in Copan UTM. Samples are stored locally at − 80 °C. When − 80 °C storage is unavailable, samples can be stored at − 20 °C before shipment to the UMCU laboratory. Samples are preferably stored in the original container and labeled with unique barcode provided by the UMCU corresponding to the INFORM RSV code. Samples are shipped frozen on dry ice to the UMCU laboratory for sequencing and culturing after each season.

### Nucleic acid extraction and RSV subtyping

Nucleic acids are extracted from 250 to 500 μL of RSV positive nasal specimens using the MagNA Pure 96 DNA and Viral NA Large Volume kit (Roche Diagnostics, Mannheim, Germany) according to the manufacturer’s instructions. Nucleic acids are eluted in 50 μL elution buffer. RSV subtyping and quantification is performed by multiplexed TaqMan RT-PCR analysis of the RSV N gene using RSV-A and RSV-B specific primer/probe mixes. The TaqMan RT-PCR reactions are performed on a StepOnePlus System (Applied Biosystems) in 10 μL total volume, including 1 μL of nucleic acids, TaqMan Fast Virus 1-Step Master Mix (Thermo Fisher Scientific), 900 nM RSV-A forward primer (5′ AGATCAACTTCTGTCATCCAGCAA 3′), 900 nM RSV-A reverse primer (5′ TTCTGCACATCATAATTAGGAGTATCAAT 3′), 300 nM RSV-B forward primer (5′ AAGATGCAAATCATAAATTCACAGGA 3′), 300 nM RSV-B reverse primer (5′ TGATATCCAGCATCTTTAAGTATCTTTATAGTG 3′), 58.3 nM RSV-A probe (5′ CACCATCCAACGGAGCACAGGAGAT 3′, 5′6-FAM/ZEN/3′IBFQ), and 66.7 nM RSV-B probe (5′ TTCCCTTCCTAACCTGGACATAGCATATAACATACCT 3′, 5′ JOE NHS/ZEN/3′ IBFQ) (Integrated DNA Technologies). Cycling conditions are 50 °C for 2 min and 95 °C for 10 min, followed by 45 cycles of 95 °C for 15 s and 60 °C for 60 s.

### RT-PCR amplification of RSV genomes and next generation sequencing

Upon RSV subtyping, the appropriate primer pairs are used to reverse transcribe and PCR amplify the four overlapping RSV genome fragments by using the SuperScript IV One-Step RT-PCR System (Invitrogen, CA) in a 9800 Fast thermal cycler (Applied Biosystems). The four overlapping genome fragments together comprise of the full RSV genome encompassing all viral genes, yet lacking the far 3′ and 5′ genome termini. Degenerate bases are used in places of genetically variable bases across RSV-A and RSV-B strains when necessary (Table 2). Cycling conditions are 55 °C for 10 min and 98 °C for 2 min, followed by 40 cycles of 98 °C for 10 s, 61 °C for 10 s and 72 °C for 3 min. Amplicons are verified on 1% agarose gels, pooled in equimolar amounts, and purified from 1% agarose gel using the GeneJet PCR Purification Kit (Thermo Fisher Scientific). The purified amplicons are then quantified using the Quant-iT PicoGreen dsDNA Assay Kit (Thermo Fisher Scientific) according to the manufacturer’s instructions. Subsequently, the normalized PCR products are subjected to Next Generation Sequencing (NGS) library construction using the Nextera XT DNA Library Prep Kit according to the manufacturer’s protocol (Illumina). Illumina sequencing adapters and barcodes are added to the tagmented DNA via PCR amplification using unique custom oligo sequences (Integrated DNA Technologies). Subsequently, the DNA is purified and size-selected using 0.6 X volume of Ampure XP reagent (Beckman Coulter, Inc.) according to the manufacturer’s protocol. Next, the purified DNA is quantified using the Quant-iT PicoGreen dsDNA Assay Kit (Thermo Fisher Scientific) and mixed in equimolar amounts. Sequencing is performed on the Illumina NextSeq500 platform (Illumina, Inc), generating paired-end 150 bp reads.

**Table 2:** Primers used in this study to amplify overlapping RSV genome fragments

Primer	Sequence (5′-3′)
RSVA-fragment 1-Fw	AAAAATGCGTAC**W**ACAAACTTGC
RSVA-fragment 1-Rev	GTTGGTCCTTGGTTTTGGAC
RSVA-fragment 2-Fw	CACAGTGACTGACAACAAAGGAG
RSVA-fragment 2-Rev	GCTCATGGCAACACATGC
RSVA-fragment 3-Fw	CGAGGTCATTGCTTGAATGG
RSVA-fragment 3-Rev	CACCACCACCAAATAACATGG
RSVA-fragment 4-Fw	AGGGTGGTGTCAAAAACTATGG
RSVA-fragment 4-Rev	ACGAGAAAAAAAGTGTCAAAAACT
RSVB-fragment 1-Fw	AAAAATGCGTACTACAAACTTGC
RSVB-fragment 1-Rev	TTGTGCTTGGCTTGTTGTTC
RSVB-fragment 2-Fw	AAGGGTTAGCCCATCCAA**M**C
RSVB-fragment 2-Rev	TGCTAAGGCTGATGTCTTTCC
RSVB-fragment 3-Fw	GTCCTCGTCTGA**R**CAAATTGC
RSVB-fragment 3-Rev	TAGGTCCTCTTTCACCACGAG
RSVB-fragment 4-Fw	GAGGGATCCACAGGCTTTAGG
RSVB-fragment 4-Rev	ACGAGAAAAAAGTGTCAAAAACT

### RSV genome assembly and annotation

Assembly of the sequencing reads into complete genomes is performed with AstraZeneca’s Next-Generation Sequencing Microbial Surveillance Toolbox (NGS-MSTB) – a fully automated distributed pipeline implemented with a Common Workflow Language (CWL), and with a user interface based on the Galaxy bioinformatics workbench [12]. The main processing step is a targeted de-novo assembly using Ariba [13] with AstraZeneca’s customized assembly protocol tuned for robustness in the presence of mixed viral subpopulations and very high coverage variability. This is followed by post-assembly filtering of the low-abundance poorly assembled quasi-species. The pipeline creates a Web report with quality control metrics and genome browser views at the contig and individual read levels. A manuscript with detailed description of the assembly pipeline and its open-source release is in preparation.

The assignment of RSV subtypes is performed during the assembly process and the assignment of RSV genotypes is performed by phylogenetic clustering using a reference database of previously described genotypes [14].

To determine the polymorphisms in the F protein binding regions of RSV mAbs, the gene sequences are translated into amino acid sequences, aligned against reference sequences (NL13 strains), and the amino acid changes are recorded.

### RSV culture

Frozen respiratory samples stored in UTM are thawed, combined 1:1 with DMEM (Dulbecco’s Minimal Essential Medium; Lonza) supplemented with 5% FBS and 100 μg/ml Normocin (InvivoGen), and subsequently filtered through a 0.45 μm filter. The filtrate is used to infect HEp-2 cells (60% confluent) in T25 flasks for 1 h at 33 °C and 5% CO2. The supernatant is replaced with fresh DMEM supplemented with 5% FBS and 100 μg/ml Normocin and placed back into the 33 °C humidified, 5% CO2 incubator. The viral culture is harvested upon reaching approximately 70% cytopathic effect (CPE) by centrifugation at 247×g for 10 min and combining the supernatant with 50% sucrose in dPBS (sterile filtered). The viruses are stored in 1 ml aliquots at − 80 °C.

### Data collection and management

Data is recorded on an electronic sample reporting form (SRF) (Table 3). SFRs from all sites are uploaded to a central database (eCASTOR) by Julius Clinical after which the clinical data are merged with the sequencing data. To ensure subject anonymity only a unique subject number and the age in months will be entered. Data will be locked after each season.

**Table 3:** Patient variables in the electronic case record form

Variables	Description
Site ID	
Study ID	
Country	
Visiting date	
Age	Age in months
Gender	Male / Female
Length of stay	< 24 h / > 24 h / Outpatient
Referring department	Paediatric Intensive Care Unit / General Paediatric Ward / Outpatient clinic (including Emergency Department)
RSV detection method	PCR / molecular point-of-care-test
RSV subtype	A / B
Storage temperature	-20 °C / -80 °C
Gestational age at birth	Calculated duration of pregnancy in weeks
Severe comorbidity	Congenital heart disease / Hematological malignancies / Neurological disease / Bronchopulmonary dysplasia / Other (specified in provided space)
Breastfeeding	Yes (exclusive) / No / Partial
Day care attendance	Yes / No
Current hay fever, asthma and/or eczema in either parent	Yes / No
Smoking in household	Yes / No
Other children in household under the age of 6	Yes / No

### Outcomes

#### Primary endpoint

RSV sequences from a global population of hospitalized children.

#### Secondary endpoints

1. Total number of RSV A and B subtypes and related genomes and the association of these subtypes with patient characteristics (Table 3)
2. Homology of the F gene from wild-type circulating RSV to that of reference strains
3. The total number of RSV strains with polymorphisms in RSV mAbs binding regions or antigenic sites of RSV F protein

### Sample size calculation

The minimal number of samples needed for this study is 2500. The sample size will result in precise frequency estimates of RSV A and B subtypes as well as polymorphisms. The width of the 95% confidence interval (CI) will be no larger than 4%. In extremely low or high prevalence (e.g. < 7.5% or > 92.5%) the width of the 95% CI will be less than 2%. This study is also well powered to detect differences in the prevalence of subtypes (RSV A vs B). An estimate of the mean prevalence of RSV A (two-thirds) was derived from the study by Zhu et al. [10]. The INFORM RSV study has at least 90% power to detect a difference in the prevalence of subtypes between groups of 7% at an alpha of 0.05 (e.g. 70% RSV A in males vs. 63% RSV A in females), and at least 90% power to detect an effect size of 0.08 using a 4 degrees of freedom chi-square test. This means, for example, that this study can detect a difference in the distribution of RSV subtypes if the prevalence of RSV A across the sites were approximately as follows: 58, 62, 66, 70, and 74%. These sample size calculations were conducted in PASS software, using the two-sided CIs for single proportions with the simple asymptotic method with continuity correction and a chi-square test power analysis. Although 2500 samples are sufficient to detect the desired effect, and based on the minimal invasiveness for INFORM participants, the study will expand and add more countries to maximize insight in geographic and temporal diversity.

## Discussion

In the INFORM RSV study, RSV isolates are subject to RSV subtyping and viral genome analysis. The main purpose of the project is to secure RSV samples to monitor RSV strains for changes in key epitopes recognized by mAbs. RSV is a member of the human orthopneumoviridae family [15], which are RNA viruses and therefore prone to genomic mutations. The possibility of immunological escape or viral resistance from mAbs approved or under development is a potential concern. In fact, a previous study performed by Regeneron (NCT02325791) to evaluate the efficacy and safety of suptavumab for the prevention of medically attended RSV infection in preterm infants failed to meet its predefined efficacy endpoint based on its reduced efficacy against RSV B strains [16]. The reduced RSV B efficacy was due to a two-amino acid change at positions 172 and 173 in the antigenic site V region of the F protein, the epitope of suptavumab, which reduced susceptibility to suptavumab neutralization in vitro. It is therefore important that clinical studies involving anti-RSV F mAbs monitor for amino acid substitutions in antigenic binding regions of RSV isolates from subjects experiencing virologic failure, and to assess the impact of these changes on phenotypic susceptibility and viral fitness.

A key challenge for the INFORM RSV study is temporal diversity, as the timing of RSV outbreaks differs by season and location around the world. Another challenge is how to best integrate and interpret whole genome sequences in relation to clinical variables. To overcome this challenge, bioinformaticians from AstraZeneca, UMCU and Julius Clinical are working closely together to develop an integrated database and a robust pipeline to characterize the thousands of RSV sequences that will be generated.

In summary, this global prospective study aims at monitoring the molecular epidemiology of RSV to ensure that already approved therapeutics and those in development will be effective against currently circulating strains worldwide. The study has the potential to provide valuable information for vaccines, monoclonal antibodies and therapeutic drugs in development and will contribute to creating an international RSV repository.

## Supplementary information

**Additional file 1: Table 1.** Countries participating in the INFORM RSV study. ^1^Two sites are collecting 50 samples each.

## Data Availability

As the current manuscript describes the study protocol and no other data, we do not have any raw data to share at the moment.

## Abbreviations

RSV: Respiratory syncytial virus
INFORM RSV: International Network for Optimal Resistance Monitoring of RSV
mAb: Monoclonal antibody
NA: Not applicable
SRF: Sample reporting form
CI: Confidence interval
F: Fusion
