# Supplementary material for: Global molecular diversity of RSV – the “INFORM RSV” study
Source: BMC Infect Dis. 2020 Jun 26;20:450. doi: 10.1186/s12879-020-05175-4 (PMC7316634; doi:10.1186/s12879-020-05175-4)
Supplement: Supplementary file 1 — Additional file 1: Table 1. Countries participating in the INFORM RSV study. 1Two sites are collecting 50 samples each. [file 12879_2020_5175_MOESM1_ESM.docx]

**Additional files**

Table 1. Countries participating in the INFORM RSV study

| **Country** | **Recruitment starting year** | **Number of targeted samples to be collected per year** | **Number of expected samples to be collected during the 5 year study period** |
| --- | --- | --- | --- |
| *Northern Hemisphere* | | | |
| The Netherlands | 2017 | 50 | 250 |
| Finland | 2017 | 50 | 250 |
| Spain | 2017 | 50 | 250 |
| The United Kingdom | 2017 | 50 | 250 |
| Japan | 2017 | 50 | 250 |
| Germany | 2018 | 50 | 200 |
| France | 2018 | 50 | 200 |
| Italy | 2018 | 50 | 200 |
| Canada | 2018 | 100^1^ | 400 |
| Russia | 2019 | 50 | 150 |
| South Korea | 2019 | 50 | 150 |
| Taiwan | 2019 | 50 | 150 |
| Mexico | 2019 | 50 | 150 |
| *Southern Hemisphere* | | | |
| South Africa | 2017 | 100 | 500 |
| Brazil | 2017 | 100 | 500 |
| Australia | 2017 | 50 | 250 |
| Chile | 2019 | 50 | 150 |
| *Total* | NA | 850 | 3,800 |

^1^Two sites are collecting 50 samples each
